# Parathyroid histology in normocalcemic and hypercalcemic primary hyperparathyroidism with hypercalciuric renal stones

**DOI:** 10.1210/jendso/bvag014

**Published:** 2026-02-23

**Authors:** Bénédicte Blanchard, Safia Hadjadj, Ellie Tang, Jennifer Kervadec, Souhila Ouchelouche, Perrine Frere, Olivier Traxer, Isabelle Wagner, Diane Evrard, Pierre Riebler, David Buob, Emmanuel Letavernier, Jean-Philippe Haymann, Caroline Halimi

**Affiliations:** INSERM, Unité Mixte de Recherche 1155, Kidney Research Centre, Hôpital Tenon, Paris 75020, France; INSERM, Unité Mixte de Recherche 1155, Kidney Research Centre, Hôpital Tenon, Paris 75020, France; INSERM, Unité Mixte de Recherche 1155, Kidney Research Centre, Hôpital Tenon, Paris 75020, France; Sorbonne Université, Paris, France; INSERM, Unité Mixte de Recherche 1155, Kidney Research Centre, Hôpital Tenon, Paris 75020, France; INSERM, Unité Mixte de Recherche 1155, Kidney Research Centre, Hôpital Tenon, Paris 75020, France; INSERM, Unité Mixte de Recherche 1155, Kidney Research Centre, Hôpital Tenon, Paris 75020, France; Sorbonne Université, Paris, France; Sorbonne Université, Paris, France; Service D’Urologie, Hôpital Tenon, Assistance Publique–Hôpitaux de Paris, Paris 75020, France; Service ORL et Chirurgie Cervico-Faciale, Hôpital Tenon, Assistance Publique–Hôpitaux de Paris, Paris 75020, France; Service ORL et Chirurgie Cervico-Faciale, Hôpital Bichat, Université Paris Cité, Assistance Publique–Hôpitaux de Paris, Paris 75018, France; Service ORL et Chirurgie Cervico-Faciale, CHU Tours, Tours 37044, France; INSERM, Unité Mixte de Recherche 1155, Kidney Research Centre, Hôpital Tenon, Paris 75020, France; Sorbonne Université, Paris, France; Service D’Anatomopathologie, Hôpital Tenon, Assistance Publique–Hôpitaux de Paris, Paris 75020, France; INSERM, Unité Mixte de Recherche 1155, Kidney Research Centre, Hôpital Tenon, Paris 75020, France; Sorbonne Université, Paris, France; Service D’Explorations Fonctionnelles, Hôpital Tenon, Assistance Publique–Hôpitaux de Paris, Paris 75020, France; INSERM, Unité Mixte de Recherche 1155, Kidney Research Centre, Hôpital Tenon, Paris 75020, France; Sorbonne Université, Paris, France; Service D’Explorations Fonctionnelles, Hôpital Tenon, Assistance Publique–Hôpitaux de Paris, Paris 75020, France; INSERM, Unité Mixte de Recherche 1155, Kidney Research Centre, Hôpital Tenon, Paris 75020, France; Sorbonne Université, Paris, France; Service ORL et Chirurgie Cervico-Faciale, Hôpital Bichat, Université Paris Cité, Assistance Publique–Hôpitaux de Paris, Paris 75018, France

**Keywords:** hypercalcemic primary hyperparathyroidism, normocalcemic primary hyperparathyroidism, calcium-sensing receptor, CYP27B1, VDR

## Abstract

**Context:**

Hypercalcemic (HPHPT) and normocalcemic primary hyperparathyroidism (NHPT) are distinct conditions with different biological and histological characteristics. Understanding their histological patterns could improve disease characterization.

**Objective:**

This study aimed to compare the histological features of NHPT and HPHPT parathyroid glands, but also rims of normal tissue, focusing on the expression of calcium-sensing receptor (CaSR), 1-α hydroxylase (CYP27B1), and vitamin D receptor (VDR).

**Methods:**

A retrospective observational study was conducted on histological and immunohistochemical data from parathyroid gland samples. The study included 50 hypercalciuric renal stone patients, of whom 18 had NHPT and 32 had HPHPT. Histological and immunohistochemical analyses were performed to evaluate cell distribution and marker expression. Parathyroid gland weight, cell distribution, and CaSR, CYP27B1, and VDR expression were analyzed and compared between NHPT, HPHPT, and rim biopsies.

**Results:**

Parathyroid gland weight and cell distribution were similar in both groups. A rim of normal tissue was more frequent in HPHPT (69% vs 37%; *P* = .02). In HPHPT, CaSR expression was decreased, while CYP27B1 and VDR expressions were increased in chief cells compared to rim tissue (*P* = .005, .004, and <.001, respectively). NHPT showed no CaSR or CYP27B1 alterations but a decreased VDR expression in oxyphil cells compared to HPHPT (*P* = .02).

**Conclusion:**

The NHPT hallmark phenotype is normal CaSR, CYP27B1/VDR expression in chief cells, with decreased VDR expression in oxyphil cells. HPHPT chief cell patterns show a marked CaSR decreased expression along with an increased CYP27B1/VDR expression, suggesting an appropriate autocrine/paracrine counterregulation to hypercalcemia/high PTH.

Primary hyperparathyroidism (PHPT) is a very common endocrine disorder in which one or more parathyroid glands are responsible for an excessive and nonadapted secretion of parathyroid hormone (PTH) in the setting of elevated or normal ionized calcium [1]. It occurs in approximately 1 in 1000 individuals in the general population, predominantly affecting women [2]. Diagnosis of primary hyperparathyroidism involves evaluating the relationship between calcium levels and PTH [3]. Histologically, PHPT commonly presents as adenoma in 85% of cases (characterized by the presence of a rim of normal tissue [4]), hyperplasia in 15% of cases, and occasionally as carcinoma [5].

Since normocalcemic primary hyperparathyroidism (NHPT) was first described in 2008 [6], it has been noticed that patients with normal calcium levels may still develop complications associated with PHPT [7, 8], reversible after treatment. While several pathophysiological mechanisms have been proposed, the specific signaling pathway responsible for normocalcemic forms of PHPT has yet to be identified [7, 9].

A few reports show more hyperplasic glands in NHPT compared to HPHPT [10]. However, function and composition of different cell types within the parathyroid glands of NHPT and HPHPT remain under investigation. Chief cells are reported to express calcium-sensing receptor (CaSR) and synthesize PTH, whereas oxyphil cells are reported to express 1-α hydroxylase (CYP27B1) and produce calcitriol, which may exert an inhibitory paracrine effect on chief cells’ PTH secretion [11]. Accordingly, an inhibitory effect of vitamin D receptor (VDR) signaling on PTH synthesis and CaSR expression was demonstrated using parathyroid-specific knockout mice [12].

Decreased CaSR expression and VDR levels have been reported in HPHPT adenoma compared to normal parathyroid tissue; however, reports are controversial regarding the localization and expression of CYP27B1 in the parathyroid glands [13-17]. In NHPT glands, a previous report shows a negative association between ionized calcium and serum PTH, suggesting that CaSR signaling would be efficient in NHPT patients [18]. Accordingly, a normal profile of CaSR with a decreased VDR expression was reported as a key feature with no data available regarding CYP27B1 expression [19].

We took advantage of a previously well-phenotype observational cohort of renal stone patients with HPHPT and NHPT [18] to characterize within adenoma/hyperplasia or normal (rim) parathyroid tissue, cell type composition, and phenotypes regarding CaSR and CYP27B1/VDR expression.

## Materials and methods

### Patients

We performed a retrospective single-center study at the University Hospital of Tenon (Assistance Publique–Hôpitaux de Paris, APHP) between June 2005 and March 2015. The study population represents a subset of the original cohort of 1671 patients previously reported in our 2024 publication [18]. A total of 58 patients underwent parathyroidectomy for PHPT; histological specimens were not available for 8 patients, who were therefore excluded, resulting in a final study population of 50 patients. The diagnosis of PHPT was assessed by the presence of a high serum ionized calcium level (>1.31 mmol/L) after an oral calcium load test associated with an inadequately suppressed PTH serum level after calcium load (ie, >30 pg/mL) as previously reported [18]. We classified the patients into 2 groups: 18 patients in the NHPT group (ionized calcium <1.31 mmol/L before calcium load) and 32 patients in the HPHPT group, all of whom had available parathyroid tissue. A total of 54 parathyroid glands were analyzed, with 2 patients with multiglandular disease.

Reports were retrieved from the archived files of patients in the Department of Physiology (Tenon Hospital, APHP) and the files of the Pathology Department (Tenon Hospital, APHP). Clinical and biological data were collected from the Department of Physiology database. Data collection was approved by the “Commission Nationale de l’Informatique et des Libertés” in accordance with French legislation (approval No. 2065902v0). All participants gave their written, informed consent.

### Parathyroid gland pathology

Adenomas are defined by the presence of a normal parathyroid tissue called rim surrounding the adenoma [4] and hyperplasia by the absence of a rim, the presence of adipocytes in the tumor, and a homogeneous parenchyma. We performed cytological and histological analysis from parathyroid gland paraffin-embedded sections of patients. Tissue blocks were sectioned at a thickness of 3 µm. We used hematoxylin-eosin staining to determine the proportion of the different cell population, namely chief cells, water-clear cells, and oxyphil cells and also the presence of a rim [20, 21].

### Parathyroid gland immunochemistry

After paraffin removal, antigen-retrieval, and incubation with a peroxidase-blocking solution, sections were incubated with one of the following primary antirabbit antibodies overnight at 4 °C: anti-PTH (Abcam catalog No. ab268111, RRID:AB_3717609, https://scicrunch.org/resolver/AB_3717609), anti-CaSR (Thermo Fisher Scientific catalog No. PA1-37213, RRID:AB_2071481, https://scicrunch.org/resolver/AB_2071481), anti-VDR (Abcam catalog No. ab3508, RRID:AB_303857, https://scicrunch.org/resolver/AB_303857), and anti-CYP27B1 (Abcam catalog No. ab206655, RRID:AB_2894966, https://scicrunch.org/resolver/AB_2894966). Sections were revealed by a secondary antirabbit antibody (N-histofine Simple Stain, Nichirei Biosciences Inc, catalog No. 424141, RRID:AB_3073750, https://scicrunch.org/resolver/AB_3073750) and then by Biotin-Free Polyvalent AEC (3-amino-9-ethylcarbazole, Dako). Negative controls were performed by omitting the primary antibody. We used normal human kidney as a positive control to ensure antibody specificity for CaSR, VDR, and CYP27B1, and PTH.

Staining was deemed positive based on specific patterns: diffuse granular cytoplasmic for PTH, membranous for CaSR, nuclear for VDR, and diffuse cytoplasmic for CYP27B1. Staining of these proteins was studied according to each cell population within the different groups. Slides stained for all antibodies were reviewed in a blinded fashion by 2 trained operators (C.H. and B.B.). Staining intensity was assessed semiquantitatively using scoring as follow: no staining:0, faint staining:1, positive staining:2, strong staining:3. Average scoring was used and a score greater than 1 was considered as a positive staining.

### Statistical analysis

Qualitative and quantitative data were reported as percentage and mean ± SD respectively. Comparison between NHPT and HPHPT glands, NHPT and rim (normal tissue adjacent to an adenoma), or HPHPT and rim was performed using the nonparametric Mann-Whitney test for quantitative data and a chi-square test for qualitative data. The results were considered statistically significant when the *P* value was less than .05. All statistical analysis were performed using Prism version 8 and StatView version 5.0.

## Results

Among our population, no statistically significant difference for age (55 ± 14.1 vs 52 ± 13.4 years) or sex (33% females vs 34% males) between the NHPT and HPHPT groups was found. As previously reported [18], comparison of biological characteristics of NHPT and HPHPT patients while performing an oral calcium load test are provided in Supplementary Table S1 [22]. Briefly, fasting ionized calcium was normal (1.27 mmol/L) in the NHPT group and high (1.40 mmol/L) in the HPHPT group (*P* < .05) with similar PTH values and calciuria, whereas after calcium load, ionized calcium was high in both groups with an inappropriate serum PTH value (high or normal and not low as expected) in both groups (52 vs 83 ng/L; *P* < .05).

**Figure 1:**
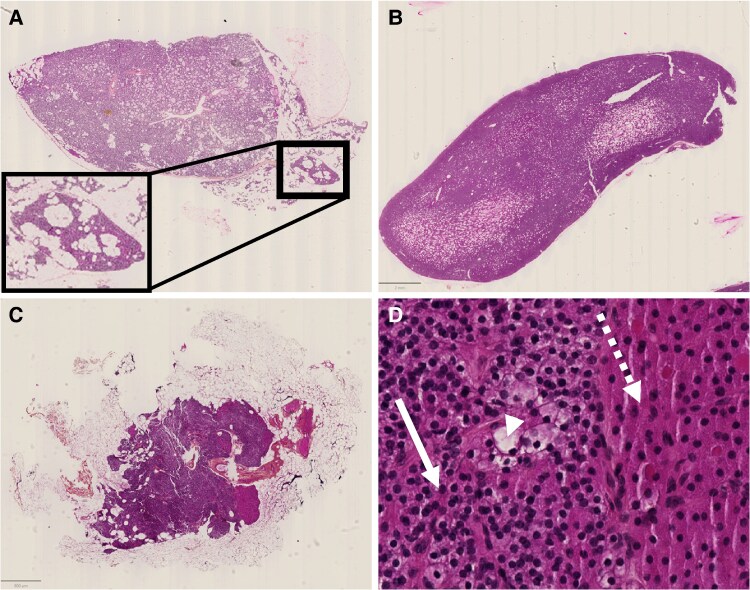
Parathyroid gland illustration of A, an adenoma in the HPHPT group with the presence of a rim (enlarged in the window); B, a hyperplasic parathyroid in the NHPT group; C, a normal parathyroid gland; and D, magnification in a normal parathyroid gland of chief cells (solid white arrow), water-clear cells (white arrowhead), and oxyphil cells (dashed white arrow). HPHPT, hypercalcemic primary hyperparathyroidism NHPT, normocalcemic primary hyperparathyroidism.

As shown in Table 1, despite similar parathyroid gland weights between the two groups, we detected a higher prevalence of a rim of normal tissue (Fig. 1) in the HPHPT compared to the NHPT group (69% vs 37% of cases, respectively; *P* < .05), thus suggesting that adenomas were predominant in the HPHPT and hyperplasia in the NHPT group. Surprisingly, there was no obvious difference in the composition of cell types between the two groups with a predominance of chief cells and only a few glands expressing a predominance of oxyphil cells. When no predominant cell-type population was present, a mixed-cell contingent (mostly chief cells and water-clear cells) was detected in a quarter to a third of cases. Prevalence of oncocytoma (ie, oxyphil cells) was also not different between the two groups (5% vs 6% in the NHPT and HPHPT groups, respectively). In normal rim tissue, chief cells were predominant with very few scattered oxyphil and water clear cells. Among 31 rims available, oxyphil cells and water-clear cells could be analyzed in only 5 and 4 cases, respectively.

**Table 1:** Comparison of parathyroid weight, prevalence of a rim, and cell-type distribution between normocalcemic primary hyperparathyroidism and hypercalcemic primary hyperparathyroidism groups

	NHPT (n = 19)	HPHPT (n = 35)	*P*
Parathyroid weight, mg	763 (±1172)	821 (±908)	.26
Presence of a rim	37%	69%	.02
Predominant chief cells	37%	54%	.22
Predominant water-clear cells	21%	14%	.52
Predominant oxyphil cells	5%	6%	.94
Mixed cells	37%	26%	.39

Abbreviations: HPHPT, hypercalcemic primary hyperparathyroidism; NHPT, normocalcemic primary hyperparathyroidism.

**Figure 2:**
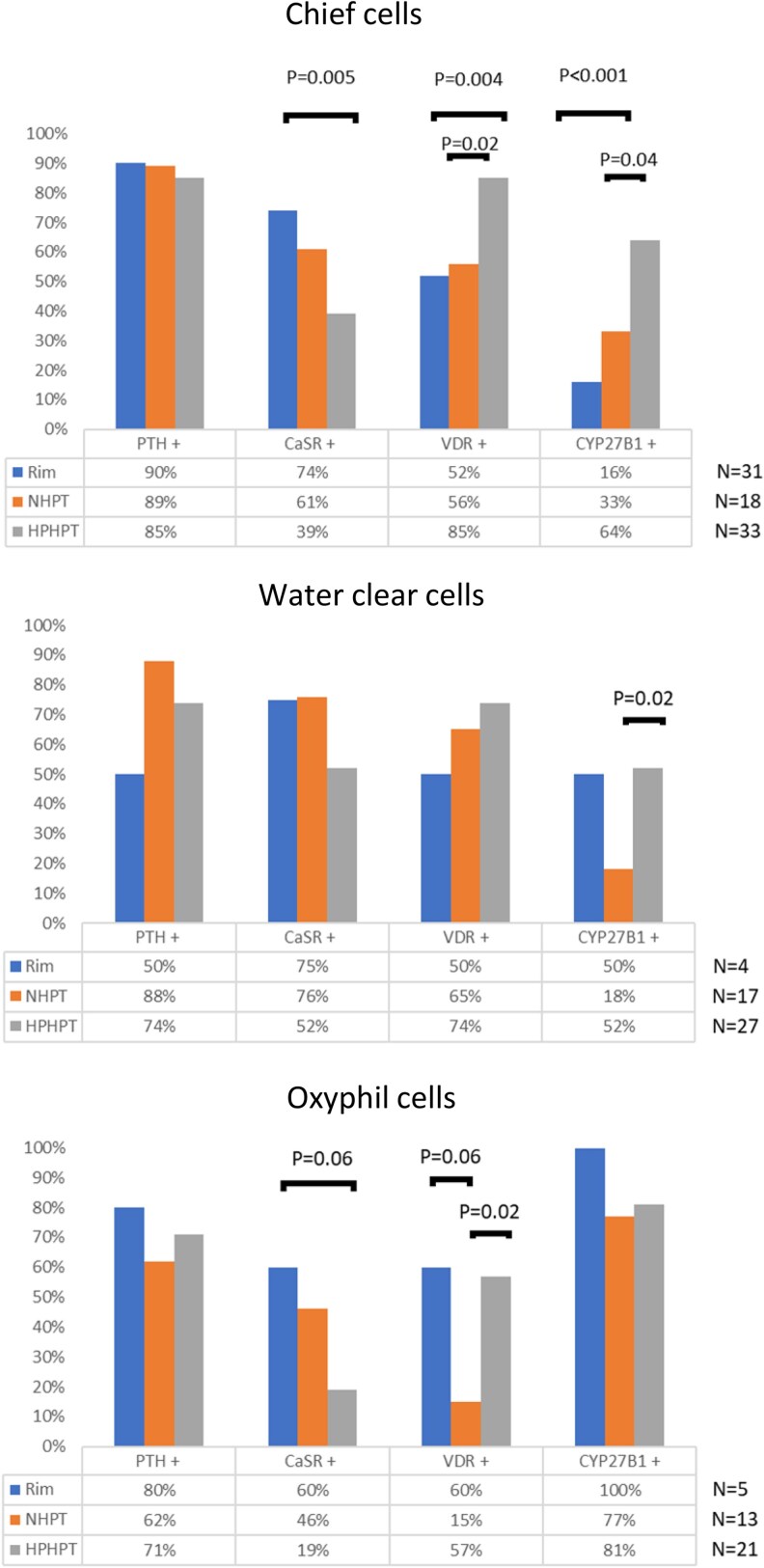
Comparison according to the different cell types (chief cells, water-clear cells, and oxyphil cells) of PTH, CaSR, VDR, and CYP27B1 expression between rim of normal tissue, NHPT and HPHPT groups. CaSR, calcium-sensing receptor staining; CYP27B1, 1-α hydroxylase staining; HPHPT, hypercalcemic primary hyperparathyroidism; NHPT, normocalcemic primary hyperparathyroidism; PTH, parathyroid hormone staining; VDR, vitamin D receptor staining.

**Figure 3:**
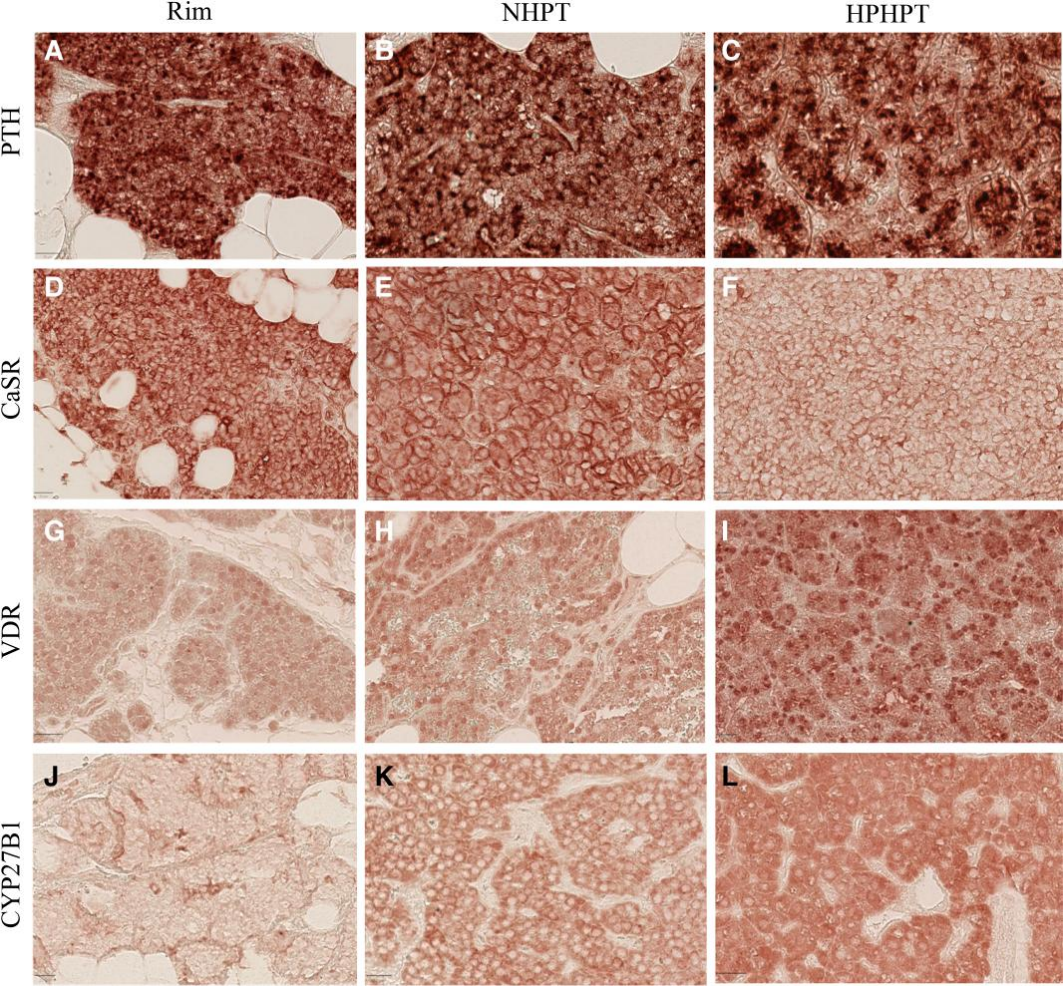
Illustration within chief cells of A to C, PTH; D to F, CASR; G to I, VDR; and J to L, CYP27B1 in a rim (A, D, G, and J), NHPT patients (B, E, H, and K), and HPHPT patients (C, F, I, and L). CaSR, calcium-sensing receptor staining; CYP27B1, 1-α hydroxylase staining; HPHPT, hypercalcemic primary hyperparathyroidism; NHPT, normocalcemic primary hyperparathyroidism; PTH, parathyroid hormone staining; VDR, vitamin D receptor staining.

**Figure 4:**
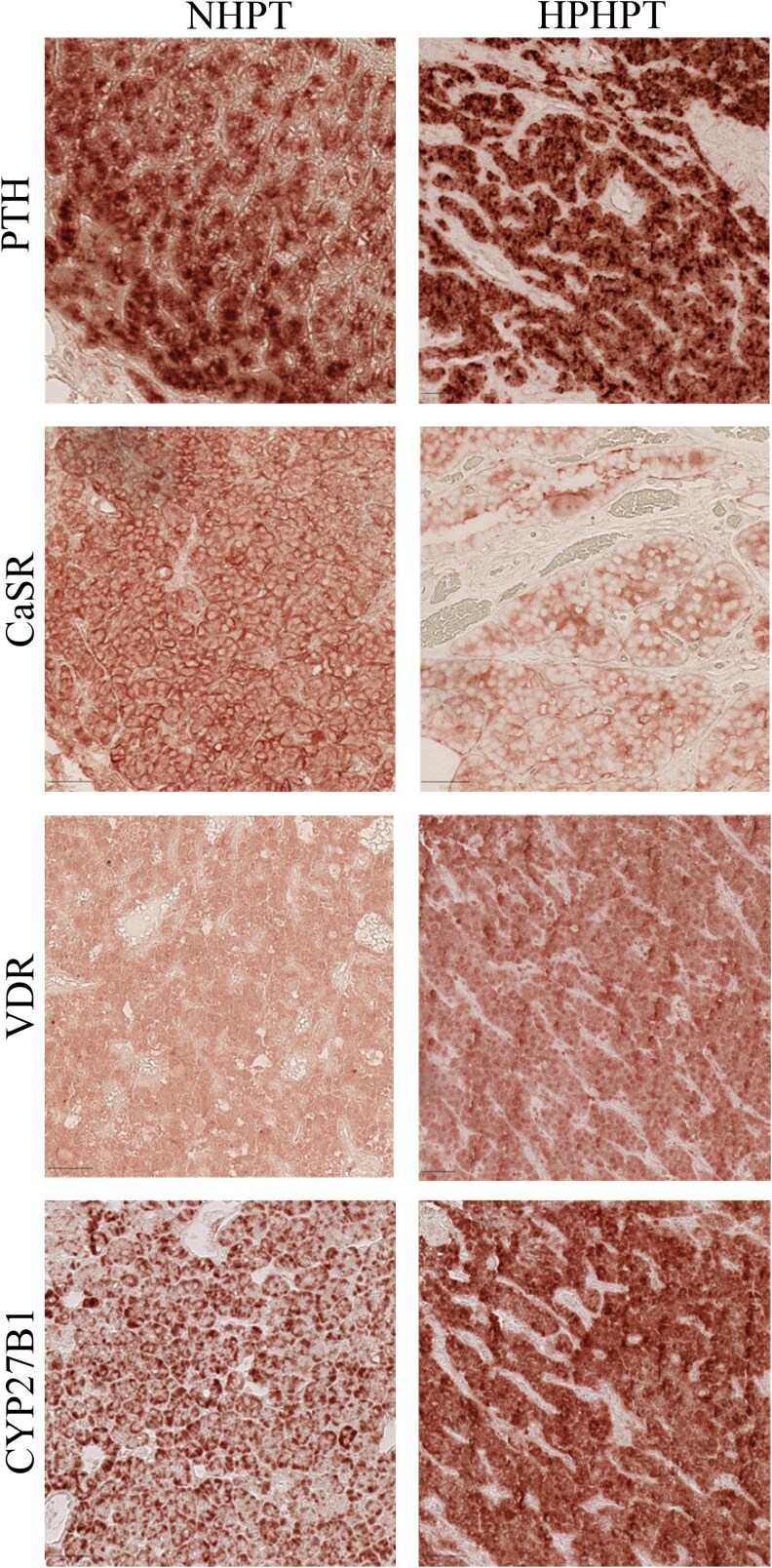
Illustration within oxyphil cells of A and B, PTH; C and D, CaSR; E and F, VDR; and G and H, CYP27B1 in NHPT patients (A, C, E, and G) and HPHPT patients (B, D, F, and H). CaSR, calcium-sensing receptor staining; CYP27B1, 1-α hydroxylase staining; PTH, parathyroid hormone staining; VDR, vitamin D receptor staining.

As shown in Figs. 2 and 3, staining for PTH was positive in normal rim tissue but also in NHPT and HPHPT glands. PTH staining was detected in all cell-type populations, including oxyphil cells (Fig. 4). Indeed, PTH staining in chief cells was present in 50% up to 90% of cases in normal rim tissue but also in the NHPT and HPHPT groups, as illustrated in Figs. 2 and 3A to 3C.

In HPHPT, CaSR expression in the chief-cell population was significantly decreased compared to normal rim tissue (39% vs 74% of cases; *P* < .05; see Figs. 2 and 3D-3F). A similar trend was noticed in oxyphil cells (see Figs. 2 and 4). Expression of VDR and CYP27B1 within the chief-cell population was significantly increased both compared to normal rim tissue and NHPT groups (see Figs. 2 and 3). Within oxyphil cells, VDR staining was also increased compared to the NHPT group (*P* = .02) but no difference was detected for CYP27B1 staining (see Fig. 2).

In the NHPT group, in all cell populations, staining for CaSR, VDR, and CYP27B1 appears similar to normal rim tissue except for a trend toward decreased VDR expression within oxyphil cells (15% vs 60%; *P* = .06; see Fig. 2). However, VDR pattern expression between the different cell types was statistically significantly different (*P* < .05) with a lower expression in oxyphil cells compared to chief cells, as shown in Fig. 2.

In normal rim tissue, a striking feature was the presence of CYP27B1 in 100% of cases within oxyphil cells and in only 16% of cases in chief cells, but similar expression for PTH, CaSR, and VDR among chief cells, water-clear cells, and oxyphil cells (as shown in Fig. 2).

## Discussion

Our study shows a higher prevalence of residual normal parathyroid tissue (rim) in the HPHPT group compared to NHPT, thus suggesting that adenomas are predominant in HPHPT whereas hyperplasia is more frequent in the NHPT group, in agreement with previous reports [10]. Cell-type distribution within parathyroid glands appears similar between the two groups. The glands of patients are predominantly composed of chief cells in both groups, while oxyphil cells are predominant in only 5% of cases. Of notice, mixed cell–type glands are composed in all cases of chief and water-clear cells, favoring the view that water-clear cells would be a differentiated state of chief cells [23 , 24 ]. If somatic tumor suppressor genes such as *MEN1* and *CDKN1B* [25] or oncogene mutations occur across various parathyroid cell types [26], we speculate that such a hypothesis is more likely to happen in HPHPT, where adenoma formation is more frequently observed. However, we cannot rule out in some cases a heterozygous mutation occurring in genes controlling cell cycle altogether with a loss of the PTH counterregulation pathway, noteworthy CaSR or CYP27B1/VDR signaling, triggering and enhancing parathyroid cell proliferation [13, 26-28]. Further investigation addressing this issue of somatic mutation of tumor suppressor genes in a nonsyndromic context is required to unravel whether clonal mechanisms may also contribute to hyperplasic parathyroid glands. Within normal rim tissue, PTH, CaSR, and VDR are expressed in chief cells but also in oxyphil cells, raising the issue of the function of oxyphil cells within normal parathyroid glands [11]. Accordingly to previous reports, oxyphil cells appear as a major source of calcitriol within the parathyroid gland [29, 30] in accordance with our data, since CYP27B1 is present in all cases in oxyphil cells but in only 16% of cases in chief cells.

Within HPHPT glands, we show decreased CaSR staining in most chief cells (and a trend within oxyphil cells) compared to parathyroid rim staining. These findings are in accordance with previous reports showing a CaSR decrease within HPHPT glands compared to normal glands, though cell types were not detailed [19, 31, 32]. Of note, CaSR expression decrease detected in most adenomas is unlikely due to high ionized calcium exposure alone as rim tissue is exposed to the same calcemia. CaSR decrease in chief cells within HPHPT supports previous data showing 1) a more frequent CaSR-decreased expression in parathyroid glands with adenoma than hyperplasia [32, 33] and 2) the reported lack of association between ionized calcium and serum PTH in HPHPT conversely to a negative association in NHPT, suggesting a preserved CaSR signaling in the latter [18]. Accordingly, our data show no significant CaSR staining decrease in NHPT group compared to normal rim tissue.

To our surprise, within chief cells, we also found in the HPHPT group a statistically significant increase of VDR and CYP27B1 staining compared to the normal rim tissue, with no obvious significant different expression within water-clear cells or oxyphil cells. We speculate that in most HPHPT cases, CaSR mediated PTH counterregulation impairment due to CaSR-decreased expression could potentially trigger both PTH secretion and parathyroid cell proliferation [34, 35]. Thus, CaSR downregulation would be either a primary cause or a secondary phenomenon to cell proliferation [27, 36]. In turn, upregulation of CYP27B1/VDR pathway signaling would act as an adaptive negative-feedback loop modulating PTH increase synthesis either directly (VDR-mediated) as suggested by Ritter et al [37] or indirectly (calcitriol mediated through the Klotho/fibroblast growth factor 23 pathway) [38]. In contrast, in the NHPT group, low VDR expression within oxyphil cells would suggest rather impaired CYP27B1/VDR upregulation, responsible for high/inappropriate serum PTH concentration, though we cannot rule out additional pathological processes including Klotho, fibroblast growth factor receptor 1, and PTH receptor, among other candidates. Further studies including genetic variants in vitamin D/1,25-hydroxyvitamin D metabolism are warranted to address this exciting issue.

Our data also provide some further evidence about the issue of a potential paracrine effect of oxyphil cells, which would synthesize in situ calcitriol acting on adjacent chief cells as previously suggested [15, 39, 40]. Indeed, CYP27B1 expression within oxyphil cells is present in up to 77% to 100% of glands, whereas within chief cells, CYP27B1 is rarely expressed in rim tissue and NHPT glands, hence suggesting a potential oxyphil cell paracrine effect as previously speculated [11]. Indeed, these data suggest that besides systemic calcitriol concentration, local parathyroid calcitriol synthesis appears as a potential key actor for an autocrine/paracrine calcitriol/VDR negative-feedback loop activation. According to this view, in HPHPT, intraglandular calcitriol concentration would be expected to be higher than in NHPT glands, in which only the number of oxyphil cells (and no or few chief cells) is the source of calcitriol production [11, 27, 41, 42].

### Limitation of the study

One main limitation of this study is the small sample size of normal rim tissue containing enough water-clear cells and oxyphil cells for analysis. Indeed, statistical power is obviously limited. Furthermore, given the number of statistical significance tests conducted in this small study, we cannot rule out the risk of false-positive findings. The conclusions drawn from our study are limited to NHPT and HPHPT patients with renal stones and hypercalciuria, and thus our results should be used and interpreted with caution when applying to potential patients with NHPT with bone involvement but without hypercalciuria and renal stones. Another limitation is the lack of normal parathyroid glands as a control group. However, using normal rim tissue as a control group provided interesting results despite a potential bias: a normal histology but in the context of hypercalcemia ± high PTH (as 69% of HPHPT were adenoma). Therefore, normal rim tissue appears as a relevant control group especially for HPHPT, whereas when compared with NHPT a potential bias may be at play as chronic exposure to elevated calcium levels may modify to a certain extent (though likely close to normal parathyroid gland) CaSR, VDR, or CYP27B1 expression within parathyroid cells.

In conclusion, our data strengthen the view that NHPT and HPHPT are distinct entities with different pathophysiological processes and signaling pathways. NHPT’s histological pattern feature appears to be characterized in most cases by parathyroid hyperplasia with a normal chief-cell phenotype and a potential dysfunctional VDR negative-feedback loop. HPHPTs are frequently adenoma, with a chief-cell pattern showing a marked CaSR-decreased expression along with a strong increased CYP27B1/VDR expression, suggesting appropriate autocrine/paracrine counterregulation. Further studies are needed to elucidate the mechanisms triggering parathyroid cell proliferation in NHPT and the likely interplay between chief cells, water-clear cells, and oxyphil cells in the control of PTH synthesis and cell proliferation in physiology and pathology. A routine analysis of parathyroid glands for CaSR, VDR, and CYP27B1 altogether with somatic mutations of genes controlling cell cycles could be of potential interest to identify patients with a high risk of hyperparathyroidism recurrence.

## Data Availability

Original data generated and analyzed during this study are included in this published article or in the data repositories listed in “References.”

## Abbreviations

APHP: Assistance Publique–Hôpitaux de Paris
CaSR: calcium-sensing receptor
CYP27B1: 1-α hydroxylase
HPHPT: hypercalcemic primary hyperparathyroidism
NHPT: normocalcemic primary hyperparathyroidism
PHPT: primary hyperparathyroidism
PTH: parathyroid hormone
VDR: vitamin D receptor
